# Comparative Analysis of Cyclin E1 Expression in Uterine Leiomyosarcomas and Leiomyomas

**DOI:** 10.3390/diseases14090324

**Published:** 2026-09-05

**Authors:** Aleksandar Rakić, Lazar Nejković, Dejan Oprić, Ana Đorđević, Marija Rakić, Aleksandar Jurišić, Danilo Obradović

**Affiliations:** 1 Obstetrics and Gynecology Clinic Narodni Front, Kraljice Natalije 62, 11000 Belgrade, Serbia; 2 School of Medicine, University of Belgrade, Dr Subotića Starijeg 8, 11000 Belgrade, Serbia; 3 Institute of Pathology, School of Medicine, University of Belgrade, Dr Subotića Starijeg 1, 11000 Belgrade, Serbia; 4 Faculty of Mathematics, University of Belgrade, Studentski trg 16, 11000 Belgrade, Serbia

**Keywords:** uterine leiomyosarcoma, leiomyoma, cyclin E1, CCNE1, immunohistochemistry, Allred score, differential diagnosis

## Abstract

**Background/Objectives:** Uterine leiomyosarcoma (uLMS) shares clinical and radiological similarities with benign uterine leiomyoma (LM), while validated discriminatory markers remain scarce. We compared cyclin E1 (CCNE1) expression in uLMS and LM tissue samples. **Methods:** This retrospective case-control study included 61 patients (16 uLMS, 45 LM) treated at a single institution (2017–2023). Of 22 eligible uLMS cases, six were excluded because suitable tissue was unavailable and they had significantly higher FIGO stages than the included cases. CCNE1 immunoreactivity was assessed using an adapted Allred score in five non-overlapping high-power fields selected from regions of highest expression. Two blinded pathologists independently scored each field, and the patient-level median was used for primary analysis. **Results:** The median CCNE1 Allred score was 6 (IQR 5–6) in uLMS versus 2 (IQR 0–3) in LM (*p* < 0.001), with excellent interobserver agreement (ICC 0.94). The AUC was 0.941 (95% CI 0.875–0.990). A data-derived cut-off ≥4 yielded 93.8% sensitivity and 84.4% specificity. A conservative minimum-expression analysis yielded an AUC of 0.882. Postmenopausal status was strongly associated with uLMS but was nearly completely confounded with the diagnostic group (12/16 uLMS vs. 0/45 LM). A premenopausal-only analysis was directionally consistent but underpowered. Clinical symptoms and tumor size did not differ between the groups. **Conclusions:** CCNE1 immunoexpression was substantially higher in uLMS than LM, with excellent reproducibility. Diagnostic-performance estimates remain exploratory and were influenced by tumor-region sampling, menopausal imbalance, and under-representation of advanced-stage uLMS. Independent multicenter validation, particularly in premenopausal and advanced-stage disease, is required before clinical application.

## 1. Introduction

Uterine leiomyosarcomas (uLMS), although comprising only 3–9% of all uterine malignancies, are characterized by an aggressive clinical course, high recurrence rates, and therapeutic resistance [1]. uLMS are rarely diagnosed preoperatively due to their clinical and radiological similarities to other uterine smooth muscle tumors, most commonly benign leiomyomas (LMs), as well as borderline entities and other benign and malignant masses [2]. Furthermore, investigations focused on improvements in the preoperative diagnosis of uLMS and identifying biomarkers to differentiate it from other uterine tumors face considerable challenges due to the tumor’s low incidence [3]. The rarity of this condition has resulted in a majority of small, diverse studies, thereby limiting the applicability of the findings [4,5].

A hallmark of cancer, particularly in aggressive subtypes, is the capacity for unlimited replication [6]. Dysregulation of the G1/S phase transition of the cell cycle is considered one of the key pathogenetic mechanisms underlying this phenomenon [7]. Numerous studies have highlighted the therapeutic potential of targeting this checkpoint, as well as the possible diagnostic utility of its key regulators [8,9]. One of the regulators is cyclin E1 (CCNE1), whose overexpression can accelerate the progression of the G1 phase [10,11]. This progression promotes uncontrolled proliferation of the cell. However, clinical evidence remains limited, and cyclin E1 immunoexpression has not been systematically evaluated using a reproducible scoring approach with independent observers and patient-level discriminatory performance analysis.

We previously conducted an integrated bioinformatics analysis of multiple Gene Expression Omnibus datasets and the Cancer Genome Atlas Sarcoma study and identified CCNE1 as a hub gene in the protein–protein interaction network of differentially expressed genes between uLMS and normal myometrium and between uLMS and LM [12]. These findings provided the rationale for protein-level validation of CCNE1 expression in clinical tissue samples.

This study aimed to compare CCNE1 expression in tissue samples of uLMS and LM, evaluate its diagnostic performance in distinguishing these entities, and identify clinical and pathological factors associated with uLMS diagnosis.

## 2. Materials and Methods

This retrospective case-control study was conducted on tissue samples obtained from patients treated for LM and uLMS at the Obstetrics and Gynecology Clinic “Narodni Front” in Belgrade, Serbia, between 1 January 2017 and 31 December 2023. Approval of the study was obtained from the Clinic Director (No. 2209-2023-023451/4) and the Ethics Committee of the Clinic (No. 2208-2023-025488). Immunohistochemical analyses, photographing of pathohistological slides, and their evaluation were performed at the Institute of Pathology, Faculty of Medicine, University of Belgrade. Approval of these analyses was granted by the Head of the Institute.

### 2.1. Participant Selection and Data Collection

This study included patients with a pathohistologically confirmed diagnosis of LM or uLMS following dilation and curettage and/or surgical treatment. We randomly selected the LM control group from the institutional pathological archive at a minimum 2:1 ratio relative to the uLMS group. The inclusion criteria for LM were histologically confirmed LM, availability of formalin-fixed paraffin-embedded tissue blocks with adequate sample quality for IHC, and surgical treatment at the same institution during the study period. We excluded cases with insufficient tissue, prior uterine malignancy, or concurrent uterine pathology.

The exclusion criteria for the whole cohort were as follows: presence of another gynecological malignancy or malignancy of other organ systems; prior radiotherapy before surgical treatment for LM or uLMS; a personal history of systemic, autoimmune, or musculoskeletal diseases, acute or chronic liver and kidney diseases; and patients with incomplete medical records regarding basic demographic data, clinical findings, and/or pathohistological reports.

The data were collected by reviewing patient medical records and included basic demographic data (age, body mass index [BMI], parity, and menopausal status), presenting symptoms, results of preoperative radiological imaging, details of the surgical procedure and intraoperative findings, and pathohistological findings. History of hormone replacement therapy or other systemic hormonal treatment was also reviewed. For uLMS, the following data were specifically recorded: disease stage according to the modified 2009 International Federation of Gynecology and Obstetrics (FIGO) classification [13], mitotic index, cytological atypia grade, presence of tumor necrosis, presence of lymphovascular invasion, and tumor type and localization. Resection margin status was not consistently available in the archived records and is not reported. For leiomyomas, tumor type and localization were recorded. Additionally, for patients with uLMS, all available follow-up data from the consultations of the Gynecological Malignancy Board of the Clinic were collected.

### 2.2. Pathohistological and Immunohistochemical Analysis

Paraffin blocks and corresponding hematoxylin and eosin (H&E)-stained slides with diagnoses of LM and uLMS from the specified period were retrieved from the archives of the Department of Pathological Anatomy of the Clinic. A pathologist from the Institute of Pathology, Faculty of Medicine, University of Belgrade, reviewed the H&E slides and selected paraffin blocks with tissue sections of adequate quality. Diagnoses were established in accordance with the WHO Classification of Tumours of the Female Genital Tract [14]. The diagnosis of uLMS was confirmed by the presence of at least two of the three key diagnostic Stanford criteria: significant cellular pleomorphism, a mitotic index of >10 mitoses per 10 high-power fields (HPF, ×400), and coagulative tumor necrosis [15]. Then, the selected paraffin blocks were examined. Blocks containing at least 5 mm^2^ of tumor tissue were included in this study. When more than one paraffin block was available for a patient, the pathologist selected the block containing the largest area of viable, well-preserved tumor tissue with minimal necrosis and technical artifacts for CCNE1 immunohistochemical staining.

Following deparaffinization and antigen retrieval, the sections were incubated with a primary antibody against cyclin E1 (clone EP126, rabbit monoclonal antibody, Bio SB, Santa Barbara, CA, USA; diluted 1:100). Immunohistochemical staining was performed manually according to the manufacturer’s instructions in an accredited reference laboratory. Testicular and placental tissues were used as external control tissues. Cell populations with known high cyclin E1 expression served as positive controls, whereas low- or negative-expressing components within the same external control tissues served as negative references for staining specificity. Specifically, seminiferous tubule epithelium showed high expression, whereas Leydig cells showed low or absent expression in testicular tissue, and decidual cells showed high expression, whereas trophoblastic cells showed low or absent expression in placental tissue. In addition, a reagent negative control was performed by omitting the primary antibody on a serial section during optimization of the staining protocol. Visualization of positive staining was achieved using a standard streptavidin–biotin technique (DAKO LSAB+ kit, Glostrup, Denmark), with diaminobenzidine (DAB) as the chromogen.

Evaluation of the immunohistochemically stained tissue sections was performed independently by two pathologists with extensive experience in gynecological pathology (D. Obradović and D. Oprić), both blinded to the final diagnosis. Immunoreactivity was quantified using the Allred score [16], originally developed and validated for hormone receptor evaluation in breast carcinoma [17], which represents the sum of a proportion score and an intensity score. The intensity score was graded from 0 to 3: 0—no expression; 1—weak intensity; 2—moderate intensity; 3—strong intensity. The proportion score was graded from 0 to 5: 0—0% positive cells; 1—<1%; 2—1–10%; 3—11–33%; 4—34–66%; 5—67–100%. The total Allred score ranged from 0 to 8. Only unequivocal nuclear staining in morphologically identifiable tumor cells was considered positive; cytoplasmic staining and staining in stromal, inflammatory, or endothelial cells were excluded from scoring. Within the tumor region(s) exhibiting the highest CCNE1 expression, as identified by the evaluating pathologist on initial low-power scanning, five non-overlapping high-power fields were selected for scoring. To assess intratumoral heterogeneity, the evaluating pathologist subsequently reviewed the entire tumor section for each case and classified CCNE1 expression as homogeneous or heterogeneous. Heterogeneous expression was defined as multifocal staining or the presence of clearly distinct areas with absent or lower CCNE1 expression within the same tumor section. In addition, the lowest-expressing tumor area(s) were identified for each patient for a complementary conservative analysis. For the conservative minimum-expression analysis, the lowest-expressing tumor area(s) identified on whole-section review were scored using the same adapted Allred criteria as in the primary analysis. In heterogeneous tumors, the lowest observed intensity score and the lowest observed proportion score within the examined section were combined to derive the conservative Allred score. In tumors with homogeneous expression, the lower of the two readers’ Allred scores was used as the conservative value. Because no validated scoring system or clinically established threshold currently exists for cyclin E1 immunoexpression in uterine smooth muscle tumors, this adapted semiquantitative system was used to integrate staining intensity and the proportion of positive tumor cells, and its use in the present study should be regarded as exploratory. For each of the five high-power fields, a combined score was calculated as the arithmetic mean of the two readers’ Allred scores, rounded to the nearest integer. The median of these five per-field combined scores was used as the primary patient-level unit of analysis. Individual reader-level and field-level scores were retained for the interobserver reliability analysis and other secondary analyses.

### 2.3. Statistical Analysis

Descriptive statistics were reported as median with interquartile range for non-normally distributed continuous variables and as absolute and relative frequencies for categorical variables. Normality of distribution was assessed using the Shapiro–Wilk test. Between-group comparisons (uLMS vs. LM) were performed using the Mann–Whitney U test for continuous variables and the Fisher’s exact test or Pearson chi-square test for categorical variables, as appropriate.

Unless otherwise specified, all CCNE1 analyses used the combined Allred score, calculated as the arithmetic mean of the two pathologists’ evaluations per high-power field and rounded to the nearest integer. The pre-specified mixed-effects ordinal regression model (intended to account for the hierarchical data structure of five nested fields per patient) failed to converge due to quasi-complete separation between the diagnostic groups. Full detail on this model and the robustness-check analysis used in its place is reported in the Supplementary Methods and Results S1.

Because this secondary field-level ordinal analysis is not the primary patient-level comparison reported in Section 3.3, its methods and results (patient-level cluster bootstrap, 2000 resamples; ridge and fixed-effects cumulative-link models) are reported in full in the Supplementary Methods and Results S1 rather than here.

The intraclass correlation coefficient (ICC) with a 95% confidence interval was calculated to quantify repeatability across the five fields sampled within a single representative block per patient within each diagnostic group. Interobserver reliability between the two pathologists was assessed at both the field level and the patient level (median Allred score) using the two-way random-effects ICC for absolute agreement and quadratic-weighted Cohen’s kappa.

Receiver operating characteristic (ROC) curve analysis was performed at the patient level using the median combined Allred score per patient, with the area under the curve (AUC), the Youden-optimal, data-derived threshold, and the corresponding sensitivity and specificity reported; 95% confidence intervals for the AUC and for threshold-based performance measures were obtained using a patient-level bootstrap (2000 resamples). As a robustness check, not an independent clinical sensitivity/specificity estimate, the same metrics were additionally computed at the individual field level. This analysis and its rationale are reported in the Supplementary Methods and Results S2. As a further robustness check, the same patient-level metrics were also computed separately for each pathologist’s individual scores to confirm that the combined-score analysis was not an artefact of the averaging procedure. These reader-specific results are reported in Supplementary Table S1.

The frequency of heterogeneous CCNE1 expression was compared between uLMS and LM using Fisher’s exact test. Conservative minimum-expression Allred scores were compared between the groups using the Mann–Whitney U test, and their exploratory discriminatory performance was assessed using the same patient-level ROC approach as in the primary analysis.

Univariate logistic regression analysis was performed to identify clinical and pathological factors associated with uLMS diagnosis. Because postmenopausal status quasi-completely separated the diagnostic groups, Firth’s penalized-likelihood logistic regression was used for all univariate and multivariate models to obtain finite, bias-reduced estimates. Variables with *p* < 0.1 on univariate analysis were entered into a multivariate model. The results are presented as odds ratios (OR) with 95% confidence intervals (CI).

Statistical analyses were performed using Python version 3.10. To ensure the robustness of the models addressing data separation and hierarchical clustering, the key results (ordinal models, Firth logistic regression, intraclass correlation coefficients, and ROC/AUC metrics) were independently cross-validated using R version 4.6.0 (R Foundation for Statistical Computing, Vienna, Austria). Statistical significance was set at a two-sided *p*-value of <0.05.

Generative AI (Claude Opus 4.8, Anthropic) was used to assist with statistical script development and execution, drafting and editing of the manuscript text, and preparation of Supplemenetary Methods and Results. The underlying study design, data collection, and interpretation of the results were performed by the authors. All AI-assisted outputs were reviewed, verified against the source data, and edited by the authors, who take full responsibility for the accuracy and content of the manuscript.

## 3. Results

### 3.1. Patient Characteristics

During the study period, 22 patients with a pathohistological diagnosis of uLMS and 50 patients with LM met the inclusion criteria. Paraffin blocks and slides suitable for CCNE1 immunohistochemistry could be retrieved from the Department of Pathology archive for 16/22 (72.7%) uLMS and 45/50 (90.0%) LM cases. For the remaining 11 cases, blocks had either been taken by the patient for supplementary analyses at another institution or had remained at the referring institution and were never transferred to the archive. The final IHC analysis cohort therefore comprised 61 patients: 16 uLMS and 45 LM (patient flow summarized in Supplementary Figure S1).

Patients with uLMS were significantly older than patients with LM (median 61 years, IQR 50.8–67.0, vs. 41 years, IQR 37.0–47.0; *p* < 0.001) and were more frequently postmenopausal (75.0% vs. 0%; *p* < 0.001). Gravidity was higher in the uLMS group (median 2.5, IQR 1.8–3.0, vs. median 1, IQR 0–2.0; *p* = 0.008), whereas parity, nulliparity, tumor size, and the presence of any preoperative symptom (abnormal bleeding, pelvic pain, rapid tumor growth, or urinary frequency) did not differ significantly between the groups (Table 1). BMI did not differ significantly between the groups (median 25.2 kg/m^2^ [IQR 20.6–28.5] in uLMS vs. 22.8 kg/m^2^ [IQR 20.2–26.8] in LM; *p* = 0.592); BMI data were available for 11 of 16 uLMS patients. Preoperative imaging suspicion of malignancy was recorded for only 15/61 patients (24.6%) and was therefore not included in the formal group comparison. None of the included patients had a documented history of hormone replacement therapy or other systemic hormonal treatment.

### 3.2. Comparison of IHC-Tested and Non-Tested uLMS Cases

Because block/slide availability, rather than random selection, determined which uLMS cases entered the IHC analysis, we compared the 16 included uLMS cases with the 6 uLMS cases that could not be tested (Table 2). The non-tested cases had a significantly higher FIGO stage: 0/6 were stage I versus 9/16 (56.2%) of the tested cases. Therefore, all the non-tested cases were stage II or higher (Fisher’s exact *p* = 0.046). The non-tested cases also trended toward larger tumors (median 140 mm, IQR 115.0–182.5 vs. 92.5 mm, IQR 57.5–111.2; *p* = 0.088) and older age (median 74 years, IQR 58.0–77.0 vs. 61 years, IQR 50.8–67.0; *p* = 0.230), though these did not reach significance at this sample size. No comparable imbalance was observed for LM (45 tested vs. 5 non-tested: age *p* = 0.627, tumor size *p* = 0.095). Because advanced-stage uLMS cases are more often referred to, or have material retained at other institutions for supplementary work-up, the IHC-tested uLMS cohort likely under-represents higher-stage disease; the diagnostic performance reported below should be interpreted with this selection toward earlier-stage, smaller uLMS in mind.

### 3.3. CCNE1 Allred Score

**Figure 1:**
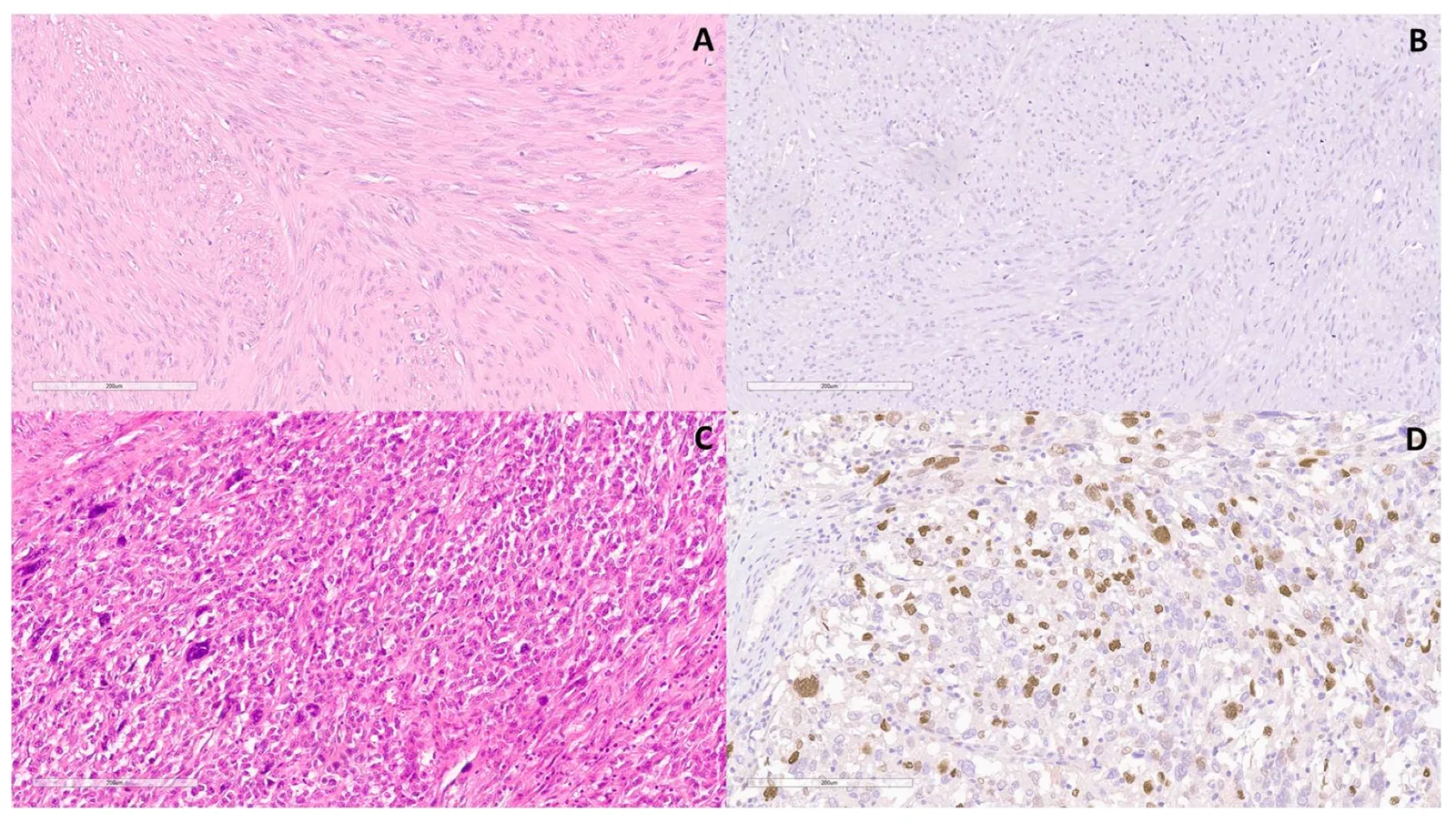
Representative photomicrographs of (**A**) leiomyoma (LM), hematoxylin-eosin stain (H&E), magnification ×200, (**B**) LM immunohistochemically stained, showing lack of cyclin E1 (CCNE1) expression, magnification ×200, (**C**) Uterine leiomyosarcoma (uLMS), H&E, magnification ×200, and (**D**) uLMS, immunohistochemically stained, showing moderate to strong CCNE1 expression in over one-third of tumor cells, magnification ×200.

**Figure 2:**
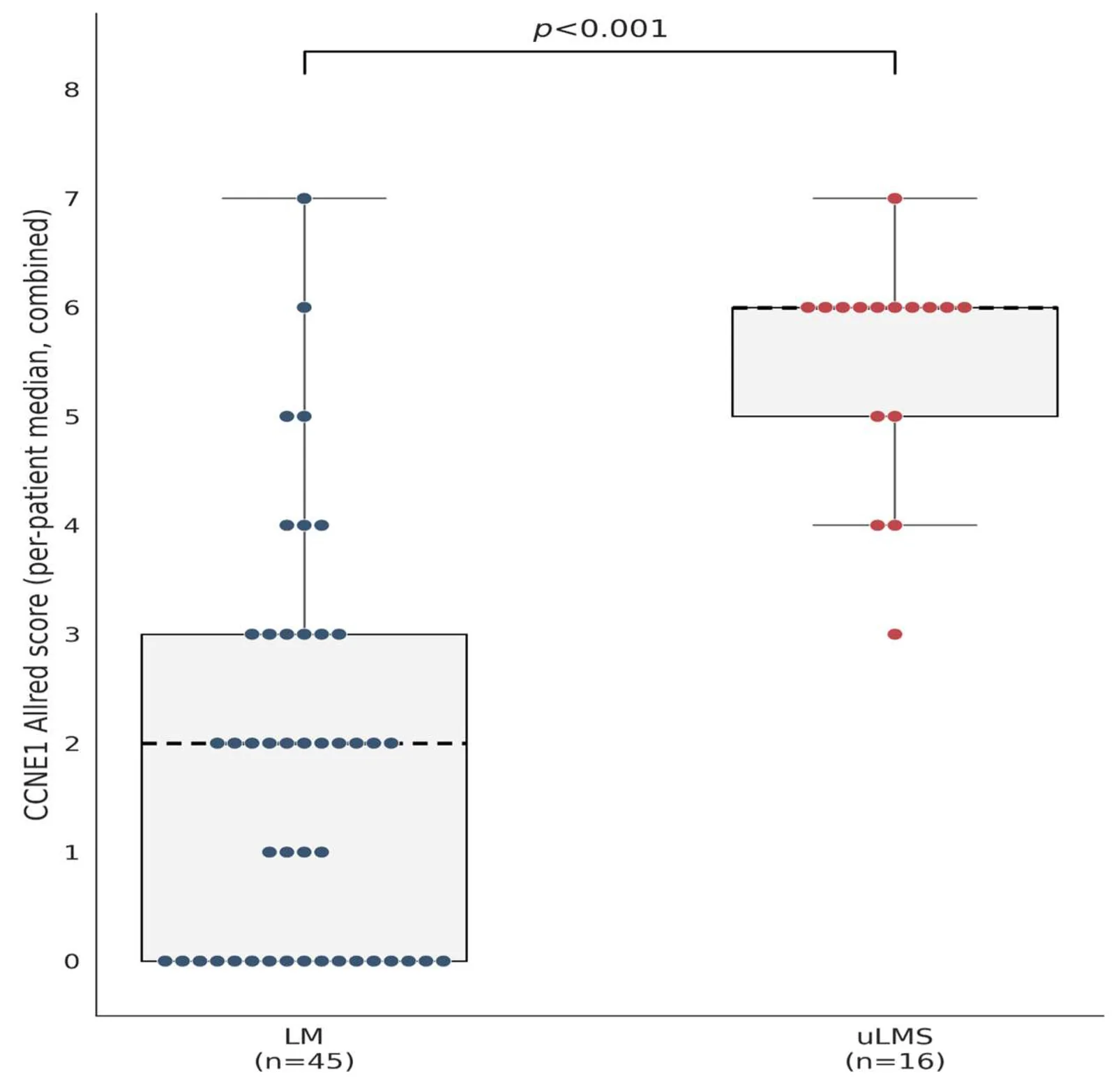
CCNE1 Allred score (per-patient median, five fields per patient) by diagnostic group.

Representative CCNE1 staining patterns in LM and uLMS are shown in Figure 1. At the patient level, the median combined Allred score (median of the 5 fields per patient) was 6 (IQR 5–6) in uLMS versus 2 (IQR 0–3) in LM (*p* < 0.001) (Figure 2). Across the five high-power fields per patient (305 field-level observations in total: 80 in uLMS, 225 in LM), the combined CCNE1 Allred scores (mean of the two readers, rounded to the nearest integer) were markedly higher in uLMS than in LM (Table 3).

The combined CCNE1 Allred category was significantly higher in uLMS than in LM. Using the patient-level cluster-bootstrap model, fields from uLMS tumors demonstrated significantly higher odds of being in a higher combined Allred category compared to LM (OR 43.4, 95% CI 13.7–331.2; *p* < 0.001). This finding was highly consistent with the original single-reader estimate (OR 41.3, 95% CI 14.0–262.6; *p* < 0.001).

Repeatability across the five sampled fields within a single representative block per patient was quantified with the one-way random-effects intraclass correlation coefficient (ICC(1,1)) within each diagnostic group, using the combined (two-reader mean) Allred scores. The field-level scores were highly consistent within each sampled block in both groups: ICC = 0.80 (95% CI 0.65–0.91) in uLMS and ICC = 0.95 (95% CI 0.92–0.97) in LM. Because all five scored fields were drawn from a single representative block per patient, this ICC primarily reflects repeatability within the sampled regions rather than heterogeneity across the tumor. Whole-section heterogeneity within the selected block was assessed separately, as described in Section 3.5. Inter-block heterogeneity was not assessed.

The interobserver reliability between the two pathologists was excellent. At the field level (*n* = 305 fields), the two-way random-effects ICC for absolute agreement was 0.93 (95% CI 0.89–0.95) and the quadratic-weighted Cohen’s kappa was 0.93; at the patient level (*n* = 61, median Allred score), the ICC was 0.94 (95% CI 0.86–0.97) and the weighted kappa was 0.94. Agreement within ±1 Allred point was 88.2% of fields. A small systematic difference was observed, with reader 1 scoring, on average, 0.34 points higher than reader 2 at the field level (95% limits of agreement −1.30 to 1.98); this offset is small relative to the between-group difference in the Allred score (approximately 4 points) and does not affect the direction or overall magnitude of the main finding.

### 3.4. ROC Curve Analysis

**Figure 3:**
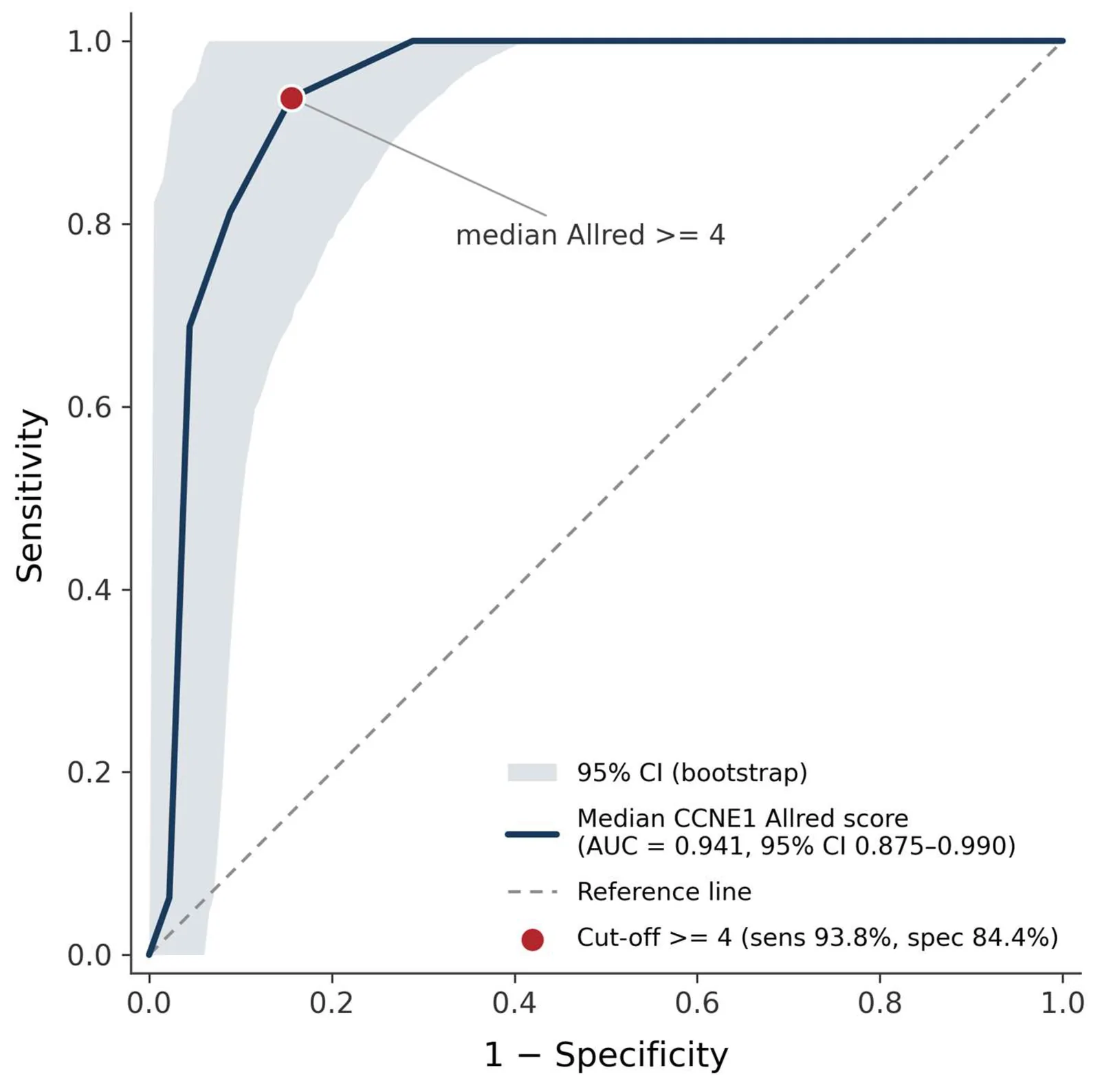
ROC curve of median CCNE1 Allred score diagnostic performance in discriminating uLMS from LM. Shaded band shows the 95% bootstrap confidence interval. Red point marks the Youden-optimal cut-off.

At the patient level, the median combined CCNE1 Allred score showed strong exploratory discriminatory performance between uLMS and LM, with an apparent area under the ROC curve (AUC) of 0.941 (95% CI 0.875–0.990, patient-level bootstrap, 2000 resamples; Figure 3). The Youden-optimal, data-derived threshold was a median Allred score ≥ 4, which yielded 93.8% sensitivity (15/16; 95% CI 76.9–100%) and 84.4% specificity (38/45; 95% CI 73.6–93.9%) (positive predictive value 68.2%, 95% CI 47.4–87.0%; negative predictive value 97.4%, 95% CI 91.2–100%; Table 4). Because this threshold was derived and evaluated in the same cohort, these figures represent apparent rather than externally validated diagnostic performance and should not yet be interpreted as a clinically validated cut-off. One uLMS patient (combined median Allred score 3) fell below this threshold; reader-specific analyses (Supplementary Table S1) showed that this same patient scored above the threshold using reader 1’s individual assessment alone, illustrating that classification at the margin is sensitive to the reader-combination method. A lower threshold of ≥3 restores 100% sensitivity at the cost of specificity (71.1%). These AUC and discriminatory-performance figures were computed in Python (scikit-learn) on the combined scores. Independent cross-validation in R (pROC) on the same combined-score data confirmed these results. Reader-specific diagnostic performance (reader 1, reader 2, and combined score individually) is reported in Supplementary Table S1 and was concordant with the combined-score analysis reported here.

### 3.5. Field-Level Robustness Check and Intratumoral Heterogeneity

As a robustness check, not an independent clinical sensitivity/specificity estimate, the same discriminatory-performance metrics were recomputed using the raw per-field combined Allred score (*n* = 305 fields: 80 uLMS, 225 LM) rather than the per-patient median; because a diagnosis is not made on a single microscopic field, the patient remains the primary unit of analysis for all diagnostic-performance claims in this manuscript. The full methods and results for this analysis are reported in the Supplementary Materials (Supplementary Methods and Results S2); in brief, field-level estimates were closely consistent with, though marginally more conservative than, the primary patient-level result above, indicating that the primary finding is not an artifact of aggregating to the per-patient median.

Heterogeneous CCNE1 expression was significantly more frequent in uLMS than in LM (9/16, 56.2% vs. 5/45, 11.1%; Fisher’s exact *p* < 0.001). Under the conservative scoring approach, the CCNE1 scores remained significantly higher in uLMS than in LM (median 4.0 [IQR 3.0–5.0] vs. 0.0 [IQR 0.0–2.0]; Mann–Whitney *p* < 0.001). The results are presented in Table 5. Exploratory diagnostic performance using the conservative minimum-expression score yielded an AUC of 0.882. At the Youden-optimal cut-off of ≥2, sensitivity was 100% (16/16) and specificity was 62.2% (28/45), compared with 93.8% sensitivity and 84.4% specificity for the primary highest-expression analysis. These findings indicate that the principal observation of higher CCNE1 expression in uLMS than in LM was not solely attributable to the field-selection strategy, although the magnitude of the estimated diagnostic Supplementary Methods and Results S2 performance varied according to the sampled tumor region. The primary highest-expression and conservative minimum-expression analyses should therefore be interpreted as complementary upper- and lower-bound estimates, respectively, while diagnostic performance under unbiased whole-tumor sampling remains to be established.

### 3.6. Predictors of uLMS Diagnosis

Univariate logistic regression identified age (OR 1.18 per year, 95% CI 1.09–1.32; *p* < 0.001), postmenopausal status (OR 252.8, 95% CI 26.1–34,484.1; *p* < 0.001), and gravidity (OR 1.88, 95% CI 1.20–3.13; *p* = 0.005) as being significantly associated with uLMS diagnosis (Table 6). Parity, nulliparity, tumor size, and individual preoperative symptoms were not significant. Because postmenopausal status quasi-completely separated 12 of the 16 uLMS patients from all 45 LM patients (0% postmenopausal), Firth’s penalized-likelihood logistic regression was used for all the univariate and multivariate models to obtain finite, bias-reduced estimates. Age, postmenopausal status, and gravidity (all *p* < 0.1 on univariate analysis) were entered into a multivariate model (*n* = 61); only postmenopausal status remained independently associated with uLMS diagnosis (OR 798.4, 95% CI 9.9–1,679,581; *p* = 0.001), while age and gravidity lost significance after adjustment, reflecting their collinearity with menopausal status. Given that only 4/16 uLMS patients were premenopausal, this odds ratio and the univariate postmenopausal-status estimate above should both be interpreted qualitatively, as confirming the direction of the association, rather than as precise effect-size estimates; even the conservative lower bound of each confidence interval indicates a strong association with uLMS diagnosis. All the univariate and multivariate estimates were independently cross-validated in R (logistf), yielding odds ratios and confidence intervals concordant with the Python (firthlogist) estimates reported above. BMI was not entered into the multivariable model because it was available for only 11/16 (68.8%) uLMS patients. Given the small uLMS sample and substantial missingness in this group, inclusion of BMI would have further reduced the analyzable sample and potentially destabilized the multivariable estimates.

### 3.7. Morphological and Clinicopathological Characteristics of the uLMS Cohort

To provide case-level clinicopathological context for the uLMS cohort, Table 7 summarizes the age, menopausal status, FIGO stage, tumor size, mitotic index, cytological atypia grade, tumor necrosis, lymphovascular space invasion (LVSI), and the patient-level combined CCNE1 Allred score for each of the 16 tested uLMS patients. Given the small number of patients, this table is presented descriptively; no formal correlation or regression analysis was performed, and no statistical claims of association between CCNE1 expression and any individual morphological feature should be inferred from it. Mitotic index, cytological atypia, tumor necrosis, and LVSI were not consistently documented at the same level of detail for every patient in the original pathology reports; where a value could not be retrieved or was not quantified in the original report, this is indicated as NR (not recorded). All 16 patients met at least two of the three Stanford diagnostic criteria for uLMS (cellular pleomorphism, mitotic index > 10/10 HPF, and coagulative tumor necrosis), consistent with the diagnostic criteria described in the Methods; this table reports the specific values underlying that classification where available.

## 4. Discussion

Our study demonstrates that CCNE1 immunohistochemical expression, quantified using an adapted, exploratory Allred score, showed strong exploratory discriminatory performance between uLMS and LM, with high sensitivity and good specificity. Practically, at this data-derived threshold, only one uLMS case in our cohort would have been missed, at the cost of misclassifying roughly one in six LM cases.

The biological rationale for this finding is established in numerous studies on malignant tumors in general, along with several studies regarding uLMS specifically. Cyclin E1, a protein encoded by the CCNE1 gene located in chromosomal region 19q12, is a key regulator of the transition from the G1 to S phases of the cell cycle. By binding to cyclin-dependent kinase 2 (CDK2), it forms an active complex that phosphorylates the retinoblastoma protein (Rb), releases transcription factors E2F, and initiates DNA replication. Under physiological conditions, CCNE1 expression is strictly time-limited and peaks at the G1/S phase boundary, after which the protein is rapidly degraded by the SCF ubiquitin ligase complex [18]. Deregulation of this checkpoint, through gene amplification, FBXW7 loss-of-function, or Rb/E2F pathway hyperactivation, produces premature and persistent S-phase entry, replication stress, and progressive genomic instability [10]. This mechanism is directly consistent with the aggressive, genomically unstable phenotype of uLMS, in which TP53 and RB1 inactivation are recurrent events. Notably, germline genome-wide association studies of uterine leiomyoma have not identified the CCNE1 locus (19q12) as a susceptibility site [19], whereas somatic genomic profiling of leiomyosarcoma has more consistently highlighted TP53/RB1/ATRX alterations [20] and CDK4 amplification [21] as recurrent cell-cycle-related events. These findings suggest that elevated CCNE1 expression in uLMS may arise predominantly through downstream Rb/E2F pathway dysregulation rather than direct CCNE1 gene amplification, although dedicated copy-number/expression correlation studies are required to test this hypothesis.

Furthermore, research by Hayashi et al. has provided additional insight into the molecular pathogenesis of uLMS through the analysis of PSMB9/β1i proteasome subunit deficiency. The study identified a direct link between this defect and cyclin E levels [22]. Their earlier experimental mouse model with targeted loss of the PSMB9/β1i gene showed spontaneous development of uLMS in approximately 37% of females by 12 months of age [23]. The proposed mechanism is that stable expression of PSMB9/β1i normally down-regulates cyclin E, while its deficiency leads to accumulation of cyclin E and defects in cell-cycle arrest, which promotes uncontrolled proliferation and chromosomal instability [22]. Hayashi et al. further proposed that total cyclin E expression levels correlate with survival and malignant behavior in uLMS. The authors also suggested a potential prognostic role analogous to that already established for cyclin E in breast cancer [22].

Our IHC findings are consistent with the existing, although scarce, immunohistochemical literature on CCNE1 in uterine mesenchymal tumors. Watanabe et al. analyzed a clinical case of a patient presenting simultaneously with LM, leiomyoma with bizarre nuclei, and uLMS within a single specimen. They investigated cyclin E1 expression as a biomarker for distinguishing between these tissue types [24]. Quantitative analysis revealed a clear gradient in the expression of this protein, where the relative value of cyclin E1 in uLMS was 103.97, more than double that of the leiomyoma variant with bizarre nuclei (40.81) and about four times that of LM (24.57) [24]. This roughly four-fold difference is consistent with our own finding of a substantially higher median Allred score in uLMS than in LM (6 [IQR 5–6] versus 2 [IQR 0–3]), although the two quantification methods are not numerically interchangeable, and the Allred score is not a ratio scale. That study, more importantly, showed that shRNA-mediated knockdown of cyclin E1 in the SK-LMS-1 uLMS cell line significantly reduced tumor growth in a xenograft model [24]. This result could be viewed as functional evidence supporting a possible role for cyclin E1 in the malignant phenotype, although it does not establish that cyclin E1 overexpression is causally responsible for malignancy in our own cases.

Earlier research by Zhai et al. [25], as well as a separate study by Hayashi et al. [26] similarly reported consistently higher cyclin E expression in uLMS than in benign smooth muscle tumors. In a larger series of 334 patients, Tanabe et al. proposed a diagnostic panel combining cyclin E1, LMP2/β1i, and Ki-67 for uLMS [27]. In other gynecologic malignancies, CCNE1 overexpression and amplification have been linked to primary platinum resistance and poor outcome in high-grade serous ovarian and endometrial carcinoma [28].

It should be noted that these studies employed different methods for interpreting CCNE1 expression, ranging from manual calculation of a positivity index to semi-quantitative categorical grading (diffuse positivity defined as over 50%, or in other studies over 80–90%, of stained cells) [25,27]. On the other hand, Watanabe et al. used digital image analysis, converting DAB-positive cells into “black dots” for automated quantification and a “merge” analysis to identify regions of cyclin E1/Ki-67 co-expression [24]. Absolute cut-off values are therefore not directly comparable across this literature, although the qualitative direction of the finding is consistent.

Several integrated bioinformatics analyses have highlighted CCNE1 as a potential marker of uLMS development, as well as a marker for differentiating uLMS from LM. Zang et al. discovered that CCNE1 was one of the major upregulated genes in uLMS and concluded that, along with FOXM1, it may participate in uLMS development [29]. In their study, Cuppens et al. applied a comprehensive integrated genomic and transcriptomic analysis on 84 uLMS samples, comprising both fresh-frozen and paraffin-embedded samples and publicly available data from The Cancer Genome Atlas (TCGA) database. Research has shown that CCNE1 is frequently amplified in uLMS, and its expression in uLMS is up to 16 times higher compared to normal myometrium [30]. Our own previous study also identified CCNE1 as one of the hub genes in uLMS [12]. Furthermore, analysis of regulatory networks has identified the microRNA miR-26b-5p as a direct regulator of this gene, while transcription factors such as Sp1, HDAC2, EP300, WT1, and TFDP1 are involved in its modulation [12]. Among these factors, WT1 and HDAC2 have independent evidence in uterine mesenchymal tumors. Nuclear WT1 expression has been investigated as a diagnostic and prognostic marker in uterine sarcomas [31,32]. On the other hand, HDAC2 belongs to the class I HDAC family that represses E2F-driven cyclin E transcription via the retinoblastoma pathway, and class I HDACs are consistently upregulated in uLMS compared to LM [33,34].

An additional finding of our study is that none of the clinical features traditionally considered suspicious for uterine sarcoma (abnormal uterine bleeding, pelvic pain, rapid tumor growth, urinary frequency) or tumor size differed significantly between uLMS and LM in this cohort, nor were they significant predictors of uLMS diagnosis on univariate analysis. Although this may partly reflect the small number of uLMS cases, this absence of a distinguishing clinical or radiological profile is itself consistent with the well-established clinical overlap between uLMS and LM that motivated this study [35,36] and underscores the diagnostic value of an objective, tissue-based marker such as CCNE1 in precisely this setting, where clinical judgment alone cannot reliably separate the two entities.

An additional finding of the present study was the substantially greater intratumoral heterogeneity of CCNE1 expression in uLMS than in LM. More than half of the uLMS cases showed heterogeneous staining across the examined tumor section, compared with a minority of LM cases. This observation is methodologically relevant because it demonstrates that CCNE1 expression in uLMS may vary considerably within the same tumor and that diagnostic performance is therefore influenced by the area selected for evaluation. Importantly, the difference between uLMS and LM persisted when the lowest-expressing tumor areas were assessed. This suggests that the principal association between higher CCNE1 expression and uLMS is not solely driven by preferential sampling of highly expressing regions. These findings further support the need for standardized sampling protocols and whole-section or multi-region evaluation in future validation studies.

The excellent agreement between the two independent observers supports the reproducibility of CCNE1 assessment using the Allred system: both the ICC and the weighted kappa were approximately 0.93–0.94 at the field and patient levels, with 88.2% of field-level readings agreeing within ±1 Allred point. The small systematic difference between the readers (0.34 points on average) had negligible influence on overall discrimination, although it did affect the classification of the one uLMS case closest to the proposed cut-off. This finding highlights that high overall reproducibility does not fully eliminate uncertainty for individual, borderline scores.

This study has several limitations. Primarily, its retrospective, single-center design and the limited number of uLMS cases reflect the rarity of the tumor, meaning our findings necessitate external validation in an independent, multicenter cohort. As noted in the Methods, the five scored fields per patient were selected from the region(s) of highest CCNE1 expression rather than from the tumor as a whole. Neither this approach nor the complementary conservative minimum-expression analysis in Section 3.5 represents unbiased whole-tumor sampling. Accordingly, the diagnostic-performance estimates should be interpreted as complementary upper- and lower-bound estimates rather than as a single unbiased point estimate. In addition, tumor hormone-receptor status was not consistently available in the retrospective records and therefore could not be incorporated into the analysis. Furthermore, tissue block availability introduced a potential selection bias; non-tested uLMS cases had a significantly higher FIGO stage than the tested cohort, suggesting the reported diagnostic performance might be optimistic for advanced-stage disease. Additionally, because our case-control design features an artificially enriched uLMS prevalence (26.2%), the positive predictive value of the CCNE1 Allred score would drop significantly in an unselected clinical setting where occult uLMS is exceedingly rare [37]. From a methodological standpoint, age and menopausal status were almost entirely confounded with the diagnostic group; Firth’s penalized-likelihood regression addresses the resulting estimation instability but does not, by itself, resolve this confounding. As proliferation-associated markers like CCNE1 may vary with hormonal status independent of malignancy, we cannot completely exclude the possibility that part of the observed difference reflects the distinct hormonal profiles of the two groups. As a targeted sensitivity check, restricting the comparison to premenopausal patients in both groups (4 uLMS vs. 45 LM, in whom menopausal status cannot confound the comparison) showed that the CCNE1 difference persisted (exact permutation test, *p* < 0.001; Supplementary Methods and Results S3), suggesting the primary finding is not solely attributable to this imbalance, though this subgroup is small, and the result should be regarded as hypothesis-supporting rather than confirmatory. Finally, while interobserver reliability was excellent, the combined score used in our primary analysis was mathematically derived rather than reaching a clinical consensus via joint slide review. Future studies should also incorporate established markers, such as Ki-67 and p53, to determine whether CCNE1 provides independent or synergistic diagnostic value.

Literature evidence that specifically addresses the role of CCNE1 in uLMS pathogenesis, or its diagnostic performance alongside established markers such as Ki-67, p53, and WT1, remains limited. Nonetheless, the growing evidence implicating CCNE1 in cell-cycle dysregulation across multiple tumor types [38], together with the results from the present study, supports the need for prospective evaluation of CCNE1 as an immunohistochemical marker in diagnostically challenging uterine smooth muscle tumors, particularly in combination with Ki-67, p53, and WT1.

## 5. Conclusions

Cyclin E1 immunoexpression, quantified using an adapted exploratory Allred score, was substantially higher in uLMS than in LM and showed excellent interobserver reproducibility. The exploratory discriminatory performance was strong, although its magnitude depended on the tumor region sampled, and the data-derived threshold should not be considered clinically validated. CCNE1 should therefore be regarded as a candidate component of future multiparametric immunohistochemical panels rather than as a standalone diagnostic test.

Generalizability was limited by two important features of this cohort. Postmenopausal status was nearly completely confounded with diagnostic group (12/16 uLMS vs. 0/45 LM), and the premenopausal-only subgroup was too small to determine whether the observed CCNE1 difference is fully independent of menopausal status. In addition, the 16 analyzable uLMS cases had significantly lower FIGO stages than the six eligible cases excluded because suitable tissue was unavailable. This leaves diagnostic performance in advanced-stage disease uncertain. Prospective, multicenter external validation including premenopausal and advanced-stage uLMS is required before clinical implementation.

## Figures and Tables

**Table 1:** Baseline demographic and clinical characteristics of the cohort.

Characteristic	uLMS (*n* = 16)	LM (*n* = 45)	*p*
Age, years, median (IQR)	61.0 (50.8–67.0)	41.0 (37.0–47.0)	<0.001
BMI, kg/m^2^, median (IQR)	25.2 (20.6–28.5) [*n* = 11]	22.8 (20.2–26.8)	0.592
Postmenopausal, *n* (%)	12 (75.0)	0 (0)	<0.001
Parity, median (IQR)	1.0 (0–2.0)	1.0 (0–2.0)	0.371
Gravidity, median (IQR)	2.5 (1.8–3.0)	1.0 (0–2.0)	0.008
Nulliparous, *n* (%)	3/16 (18.8)	17/45 (37.8)	0.222
Tumor size, mm, median (IQR)	92.5 (57.5–111.2)	80.0 (60.0–100.0)	0.500
Any preoperative symptom, *n* (%)	13/16 (81.2)	35/45 (77.8)	1.000
Abnormal bleeding, *n* (%)	10/16 (62.5)	24/45 (53.3)	0.572
Pelvic pain, *n* (%)	4/16 (25.0)	14/45 (31.1)	0.757
Rapid tumor growth, *n* (%)	2/16 (12.5)	6/45 (13.3)	1.000
Urinary frequency, *n* (%)	0/16 (0)	3/45 (6.7)	0.560

**Table 2:** Comparison of uLMS cases included in vs. excluded from the IHC analysis.

Characteristic	Tested (*n* = 16)	Non-Tested (*n* = 6)	*p*
Age, years, median (IQR)	61.0 (50.8–67.0)	74.0 (58.0–77.0)	0.230
Tumor size, mm, median (IQR)	92.5 (57.5–111.2)	140.0 (115.0–182.5)	0.088
FIGO stage I, n/N (%)	9/16 (56.2)	0/6 (0)	0.046

**Table 3:** Distribution of field-level CCNE1 Allred scores by diagnostic group.

Allred Score	uLMS Fields, *n* (%) (*n* = 80)	LM Fields, *n* (%) (*n* = 225)
0	0 (0)	93 (41.3)
1	0 (0)	19 (8.4)
2	2 (2.5)	50 (22.2)
3	3 (3.8)	24 (10.7)
4	11 (13.8)	20 (8.9)
5	11 (13.8)	8 (3.6)
6	47 (58.8)	6 (2.7)
7	5 (6.2)	3 (1.3)
8	1 (1.2)	2 (0.9)

**Table 4:** Diagnostic performance of patient-level median CCNE1 Allred score for uLMS vs. LM.

Metric	Value
AUC (95% CI)	0.941 (0.875–0.990)
Optimal cut-off (Youden index)	median Allred score ≥ 4
Sensitivity	93.8% (15/16; 95% CI 76.9–100%)
Specificity	84.4% (38/45; 95% CI 73.6–93.9%)
Positive predictive value	68.2% (95% CI 47.4–87.0%)
Negative predictive value	97.4% (95% CI 91.2–100%)
True positive/False negative	15/1
True negative/False positive	38/7

Predictive values (PPV, NPV) are specific to this case-control cohort’s enriched uLMS prevalence and are not directly generalizable (see Limitations).

**Table 5:** Intratumoral heterogeneity and conservative minimum-expression CCNE1 Allred score, uLMS vs. LM.

Characteristic	uLMS (*n* = 16)	LM (*n* = 45)	*p*
Conservative minimum-expression Allred score, median (IQR)	4.0 (3.0–5.0)	0.0 (0.0–2.0)	<0.001
Heterogeneous CCNE1 expression, n/N (%)	9/16 (56.2)	5/45 (11.1)	<0.001

**Table 6:** Univariate and multivariate Firth logistic regression for factors associated with uLMS diagnosis.

Variable	Univariate OR (95% CI)	*p*	Multivariate OR (95% CI)	*p*
Age (per year)	1.18 (1.09–1.32)	<0.001	0.93 (0.77–1.10)	0.395
Postmenopausal status	252.8 (26.1–34,484.1)	<0.001	798.4 (9.9–1,679,581)	0.001
Gravidity	1.88 (1.20–3.13)	0.005	1.59 (0.58–5.70)	0.358
Parity	1.29 (0.70–2.39)	0.420	–	–
Nulliparity	0.42 (0.10–1.46)	0.180	–	–
Tumor size (per mm)	1.007 (0.998–1.021)	0.113	–	–
Abnormal bleeding	1.42 (0.46–4.61)	0.545	–	–
Pelvic pain	0.78 (0.21–2.60)	0.696	–	–
Rapid tumor growth	1.05 (0.18–4.69)	0.954	–	–
Urinary frequency	0.37 (0.00–4.11)	0.468	–	–

The univariate and multivariate odds ratios for postmenopausal status have extremely wide confidence intervals due to quasi-complete separation (0% postmenopausal in the LM group). Firth’s penalized-likelihood regression provides finite, bias-reduced point estimates under this separation but does not resolve the underlying confounding between menopausal status and diagnostic group (see Limitations and Supplementary Methods and Results S3). These two estimates should be interpreted qualitatively, as confirming the direction of the association, rather than as precise effect-size estimates.

**Table 7:** Morphological and clinicopathological characteristics of the 16 tested uLMS patients.

Patient	Age	Postmenopausal	FIGO Stage	Size (mm)	Mitotic Index (/10 HPF)	Atypia	Necrosis	LVSI	Cyclin E1 (Allred)
uLMS-01	35	No	IIB	115	20	Severe	Present	Present	6
uLMS-02	38	No	IIIB	60	30	Severe	Present	Present	5
uLMS-03	41	No	IB	20	>10	Mild	Present	Absent	5
uLMS-04	44	No	IIA	90	26	Severe	Present	Present	6
uLMS-05	53	Yes	IVB	180	22	Severe	Absent	Absent	7
uLMS-06	54	Yes	IIA	45	>10	Severe	Present	Absent	6
uLMS-07	57	Yes	IB	110	14	Severe	Present	Absent	6
uLMS-08	60	Yes	IB	100	36	Severe	Absent	Present	6
uLMS-09	62	Yes	IIB	430	46	Severe	Present	Present	4
uLMS-10	62	Yes	IB	95	6	Moderate	Present	Absent	4
uLMS-11	65	Yes	IB	110	30	Severe	Present	Absent	6
uLMS-12	67	Yes	IA	50	5	Severe	Present	Absent	6
uLMS-13	67	Yes	IB	60	NR	Severe	Present	Absent	3
uLMS-14	67	Yes	IA	35	16	Severe	Present	Absent	6
uLMS-15	76	Yes	IVB	180	>10	Moderate	Present	Absent	6
uLMS-16	81	Yes	IB	85	13	Moderate	Present	NR	6

NR = not recorded in the original pathohistological report. Atypia was graded descriptively as mild, moderate, or severe (1, 3, and 12 cases, respectively), as reported by the evaluating pathologist. Cyclin E1 (Allred) = patient-level median combined Allred score across five fields. This table is descriptive only; no formal statistical association between CCNE1 expression and individual morphological features was tested given the small sample size.

## Data Availability

The data presented in this study are available upon request from the corresponding author due to restrictions arising from the institutional ethics committee approval, which authorizes use of the data by the study investigators but not public deposition or third-party redistribution.

## Supplementary Materials

The following supporting information can be downloaded at: https://www.mdpi.com/article/10.3390/diseases14090324/s1, Figure S1: STARD-style patient flow diagram; Table S1: Reader-specific (reader 1, reader 2, combined) patient-level diagnostic performance; Supplementary Methods and Results S1: ordinal mixed-effects modeling of field-level CCNE1 Allred scores; Supplementary Methods and Results S2: the field-level robustness analysis; Supplementary Methods and Results S3: the premenopausal-subgroup sensitivity analysis for age/menopause confounding.

## Abbreviations

The following abbreviations are used in this manuscript:

uLMS	uterine leiomyosarcoma
LM	leiomyoma
CCNE1	cyclin E1
IHC	immunohistochemistry
H&E	hematoxylin and eosin
HPF	high-power field
ROC	receiver operating characteristic
AUC	area under the curve
CI	confidence interval
IQR	interquartile range
OR	odds ratio
ICC	intraclass correlation coefficient
FIGO	International Federation of Gynecology and Obstetrics
PPV	positive predictive value
NPV	negative predictive value
CDK2	cyclin-dependent kinase 2
Rb	retinoblastoma protein
RB1	retinoblastoma 1 (gene)
E2F	E2F transcription factor family
TP53	tumor protein p53 (gene)
FBXW7	F-box and WD repeat domain-containing 7
SCF	Skp1-Cullin1-F-box (ubiquitin ligase complex)
FOXM1	forkhead box M1
WT1	Wilms tumor 1
HDAC2	histone deacetylase 2
EP300	E1A-binding protein p300
TFDP1	transcription factor Dp-1
PSMB9	proteasome subunit beta type-9
TCGA	The Cancer Genome Atlas
DAB	3,3′-diaminobenzidine
DNA	deoxyribonucleic acid
