# Supplementary material for: Comparative Analysis of Cyclin E1 Expression in Uterine Leiomyosarcomas and Leiomyomas
Source: Diseases. 2026 Sep 5;14(9):324. doi: 10.3390/diseases14090324 (PMC13604967; doi:10.3390/diseases14090324)
Supplement: Supplementary file 1 [file diseases-14-00324-s001.zip › diseases-4513448-supplementary.pdf]

Supplementary Materials

Comparative analysis of cyclin E1 expression in uterine leiomyosarcomas and leiomyomas

Figure S1. Patient flow diagram.

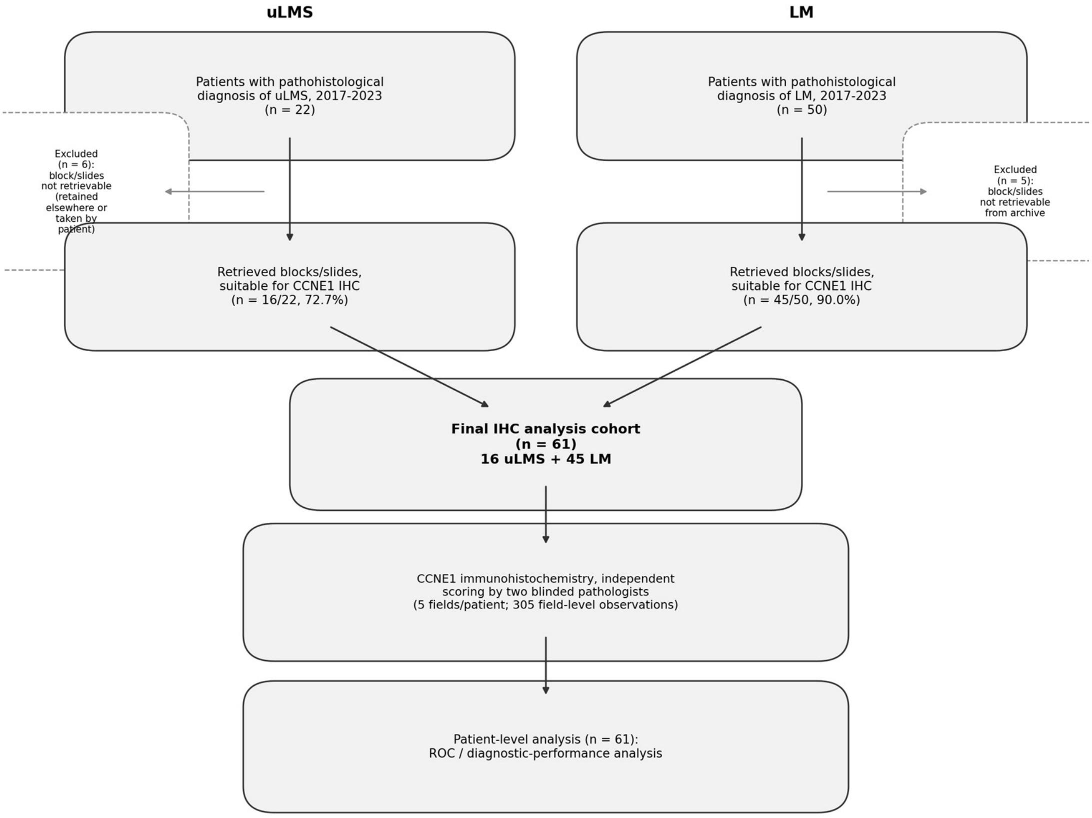

STARD-style flow diagram showing patient identification, block/slide retrieval, and the final immunohistochemistry analysis cohort for uterine leiomyosarcoma (uLMS) and leiomyoma (LM).

Table S1. Reader-specific diagnostic performance.

Patient-level diagnostic performance (median Allred score per patient across five fields) computed separately for each pathologist and for the combined (two-reader mean) score, at the exploratory, data-derived threshold of median Allred score ≥ 4. These analyses assess the robustness of the combined-score findings and illustrate the degree of reader-related variability at the exploratory threshold.

Analysis	AUC	Sensitivity	Specificity
Reader 1	0.947	100.0% (16/16)	84.4% (38/45)
Reader 2	0.922	81.2% (13/16)	84.4% (38/45)
Combined (two-reader mean)	0.941	93.8% (15/16)	84.4% (38/45)

Supplementary Methods and Results S1: Ordinal mixed-effects modeling of field-level CCNE1 Allred scores.

To assess whether the field-level (rather than patient-level) association between diagnostic group and CCNE1 Allred category was robust to an alternative modeling strategy, we additionally fit cumulative-link (proportional-odds) ordinal regression models to the field-level data (n = 305 fields within 61 patients). A mixed-effects model with a random intercept per patient failed to converge for the single-reader data (gradient and Hessian condition-number diagnostics indicated near-complete separation at the patient-cluster level). It also failed to converge for the combined-score data. As an alternative, we implemented a patient-level cluster bootstrap (2,000 resamples): an L2-penalized (ridge) proportional-odds model was applied to the single-reader data, and an unpenalized fixed-effects cumulative-link model was applied to the combined-score data, in each bootstrap resample, to obtain cluster-robust confidence intervals for the odds ratio.

Fields from uLMS tumors demonstrated significantly higher odds of being in a higher combined Allred category compared to LM (OR 43.4, 95% CI 13.7–331.2; p<0.001), consistent with the single-reader estimate (OR 41.3, 95% CI 14.0–262.6; p<0.001). These field-level ordinal estimates are directionally and qualitatively consistent with the primary patient-level result (median Allred score comparison, Results Section 3.3) and are reported here as a robustness check rather than as an independent primary finding, given the complexity of the modeling approach relative to the primary patient-level comparison.

Supplementary Methods and Results S2: Field-level robustness analysis.

As a robustness check, the same discriminatory-performance metrics reported in the main text (Section 3.4) were recomputed using the raw per-field combined Allred score (n = 305 fields: 80 uLMS, 225 LM) rather than the per-patient median. This is not an independent clinical sensitivity/specificity estimate, because a diagnosis is not made on the basis of a single randomly selected field. It is reported only to show that the patient-level result is not driven by the choice of the per-patient median as the summary statistic. Using the same patient-level cluster bootstrap (2,000 resamples) to account for the non-independence of fields from the same patient, the field-level apparent AUC was 0.933 (95% CI 0.866–0.984). At the same exploratory threshold (≥ 4), 75/80 (93.8%) uLMS fields and 186/225 (82.7%) LM fields were classified concordant with the field-level diagnostic group. These field-level estimates are closely consistent with, though marginally more conservative than, the primary patient-level result, indicating that the primary finding is not an artifact of aggregating to the per-patient median. The patient (not the field) remains the primary unit of analysis for all diagnostic-performance claims in the main text.

Supplementary Methods and Results S3: CCNE1 Allred score in the premenopausal subgroup (age/menopause-confounding sensitivity analysis).

Postmenopausal status quasi-completely separated the diagnostic groups in the main cohort (12/16 uLMS postmenopausal vs. 0/45 LM postmenopausal), so age- and menopause-related differences cannot be fully excluded as contributors to the observed CCNE1 difference between uLMS and LM (see Limitations). As a targeted sensitivity analysis, we restricted the comparison to the subset of patients who were premenopausal in both diagnostic groups, in whom menopausal status cannot confound the group comparison: 4 uLMS and 45 LM patients. Patient-level combined Allred scores were 5, 5, 6, and 6 in the four premenopausal uLMS patients, compared with a median of 2 (IQR 0-3) in the 45 premenopausal LM patients; all four uLMS values exceeded the LM median and fell at or above the 95th percentile of the LM distribution.

Because of the marked disparity in ties and group sizes (n=4 vs. n=45), the comparison was evaluated with an exact permutation test on the rank-sum statistic with mid-rank tie correction (211,876 possible group-label permutations enumerated exactly), rather than the default asymptotic normal approximation, which is not reliable at this sample size (two-sided exact permutation test, p=0.0003). Although this subgroup is small and the result should be interpreted as hypothesis-supporting rather than confirmatory, the direction and magnitude of the CCNE1 difference between uLMS and LM persisted when menopausal status could not confound the comparison, suggesting that the primary finding is not solely attributable to the age/menopausal-status imbalance between groups. Firth's penalized-likelihood regression (used elsewhere in this manuscript to obtain finite estimates under quasi-complete separation) addresses separation-induced estimation instability only; it does not, by itself, address confounding, and this premenopausal-subgroup comparison, not the Firth model, is the relevant evidence for that specific question.
